# Inhibition of Crystal Nucleation and Growth in Aqueous Drug Solutions: Impact of Different Polymers on the Supersaturation Profiles of Amorphous Drugs—The Case of Alpha-Mangostin

**DOI:** 10.3390/pharmaceutics14112386

**Published:** 2022-11-05

**Authors:** Arif Budiman, Zahra Ganesya Citraloka, Muchtaridi Muchtaridi, Sriwidodo Sriwidodo, Diah Lia Aulifa, Agus Rusdin

**Affiliations:** 1 Department of Pharmaceutics and Pharmaceutical Technology, Faculty of Pharmacy, Universitas Padjadjaran, Jl. Raya Bandung-Sumedang Km. 21, Bandung 45363, Indonesia; 2 Department of Pharmaceutical Analysis and Medicinal Chemistry, Faculty of Pharmacy, Universitas Padjadjaran, Jl. Raya Bandung-Sumedang Km. 21, Bandung 45363, Indonesia; 3 Department of Pharmacy, Poltekkes Kemenkes Bandung, Jl. Prof. Eyckman No. 24, Bandung 40161, Indonesia

**Keywords:** supersaturation, polymers, nucleation, crystallization, alpha-mangostin

## Abstract

The polymer used in supersaturated solutions plays a critical role in maintaining supersaturation levels of amorphous drugs. The prevention of drug crystallization in the supersaturated solutions by adding polymers depends on their ability to inhibit nucleation and crystal growth of drugs. This showed that understanding the mechanism of nucleation inhibition by polymers is necessary to develop the drug formulation in supersaturated solutions. Therefore, this study aims to evaluate the impact of water-soluble polymers on the supersaturation behavior of drugs and elucidate the mechanism of maintaining the supersaturation levels in an aqueous solution. It was carried out using alpha-mangostin (AM) as a model of the poorly water-soluble drug, while hypromellose (HPMC), polyvinylpyrrolidone (PVP), and eudragit were used as polymers. Their ability to inhibit the nucleation and crystal growth of AM was also evaluated. The supersaturation profiles of AM were measured in biorelevant dissolution media, while the crystal growth rate of AM was evaluated from the decrease in dissolved drug concentration by determining the induction time for AM nucleation. The interaction of AM with each polymer was evaluated and predicted by FT-IR, NMR measurement, and an in silico study, respectively. Based on observation, the PVP effectively maintained AM in a supersaturated state for the long term while eudragit conserved for 15 min. Meanwhile, an inhibitory effect of HPMC on the AM crystal nucleation was not observed. It was also discovered that the effectiveness of the various polymers depends on the interaction between the polymer and the drug. FT-IR and in silico studies demonstrated that the interaction of PVP-AM had the best polymer compared to eudragit and HPMC. NMR analysis suggested that the interaction between the methyl group from PVP with the carbonyl group of AM occurred in the PVP solution. The viscosity measurement revealed that the inhibition of nucleation and crystal growth of AM was not caused by increasing the viscosity. These results indicated that polymer–AM interactions could contribute to the crystallization inhibition and maintenance of AM in a supersaturated state. Therefore, an investigation of the mechanism of drug nucleation inhibition by polymers is recommended in the selection of crystallization inhibitors and a planned strategy to develop supersaturated formulations of drugs.

## 1. Introduction

Almost 84% of drug products worldwide that are administered orally require appropriate delivery and absorption for the treatment of diseases [1]. Meanwhile, the aqueous solubility of a drug in an oral delivery system also influences its bioavailability because drug molecules can be absorbed after dissolving in gastrointestinal fluid [2,3]. Therefore, it is necessary to develop a strategy to improve drug solubility in the formulation of poorly water-soluble drugs. This is because approximately 75% of the drug development candidates are insoluble in water and belong to biopharmaceutical classification system (BCS) classes II and IV [4]. Several strategies for improving the solubility of poorly water-soluble drugs have been studied and evolved over a period. These include reduction in particle size and increased surface area (drug nanosizing), salt formation, solubilization of drug in co-solvents or micellar solutions, inclusion complex such as cyclodextrins or encapsulation into lipid-based nanocarrier (liposome) [5]. These systems can protect the drug-sensitive to environmental effects, the cell membrane, expedite drug action, improve treatment efficiency, and control drug delivery to a specific point in the body [2]. 

Amorphous drugs with high Gibbs free energy are among the most promising formulations to improve drug delivery due to their ability to generate supersaturated aqueous solutions of poorly water-soluble drugs [6,7]. In the supersaturated state, the amount of drug crossing the biological membrane will be improved because the thermodynamic activity of a drug is enhanced beyond its solubility [8]. The amorphous drug in the supersaturation state is thermodynamically unstable, which leads to nucleation and crystallization of the poorly water-soluble drug. Therefore, the additive is very important to inhibit nucleation and crystallization, maintain a high degree of supersaturation during the intestinal transit time, and satisfactorily improve oral drug absorption [9,10,11].

The inhibitory effects of additives on drug crystallization in supersaturated solutions have been investigated based on the inhibition of crystal nucleation and crystal growth of the drug [12,13]. In the solution system, nucleation plays a critical role in determining the final crystal properties, which makes it important in pharmaceutical systems. The drug delivery systems using supersaturated solution formulations will be a challenge in the development of orally administered formulations because the nucleation mechanisms and kinetics of the drug in the solution affect the achievable supersaturation level during the intestinal transit time [11]. Therefore, understanding the mechanism of nucleation kinetics and the crystallization inhibition by additives is necessary to optimize the control crystallization of drugs from supersaturated solutions. 

Although the crystallization inhibition of drugs in the aqueous supersaturated solutions by polymeric additives has been extensively investigated [13,14,15], the mechanism of crystallization inhibition for each drug is different because of the individual’s specific physicochemical properties [11]. The maintenance of a high degree of drug supersaturation by polymeric additives depends on their ability to inhibit nucleation and crystal growth. In this study, the inhibition mechanism of drug crystal nucleation by different polymers based on a molecular-level characterization in the supersaturated solution was evaluated. Alpha-mangostin (AM) was used as a model of the poorly water-soluble drug because it is an active compound sourced from herbal plants, which has poor aqueous solubility and low oral bioavailability [16]. Meanwhile, hypromellose (HPMC) [17,18], polyvinylpyrrolidone (PVP) [19,20], and eudragit were used as polymers due to their ability to inhibit drug crystallization in the supersaturated solutions based on previous reports [10]. The crystallization inhibition of AM was also investigated using water-soluble chitosan as a polymer in pure water, as reported in our previous study [21]. This is because water-soluble chitosan was not soluble in the buffer/biorelevant dissolution media. The inhibition mechanism of the crystal nucleation of AM by the polymer in the supersaturated solutions using biorelevant dissolution media has not been studied. There is also no report on the use of HPMC, PVP, and eudragit to inhibit the crystallization of AM, as well as the relationship between AM-polymer interactions and their molecular mobility with crystallization inhibition of AM in the supersaturated system. Therefore, an investigation of the ability of each polymer to inhibit the crystallization of AM in supersaturated solutions using biorelevant dissolution media is necessary to develop the formulation of AM as poorly water-soluble drugs, specifically for oral drug formulation.

## 2. Materials and Methods

### 2.1. Materials

**Figure 1:**
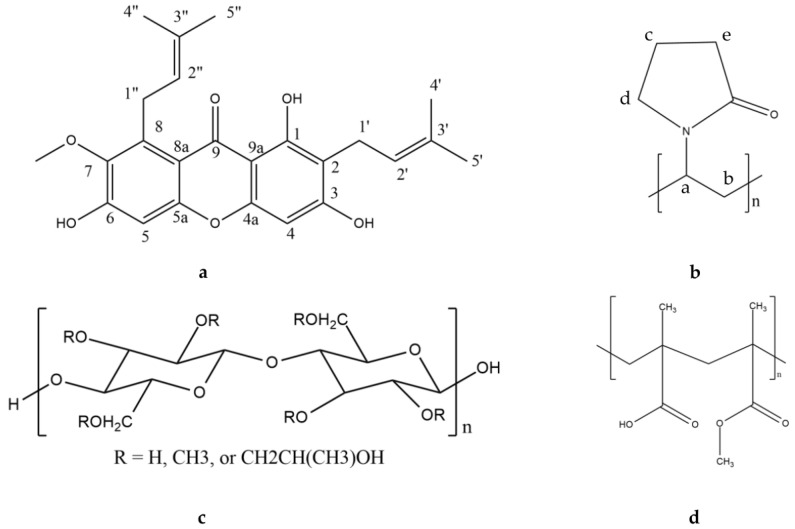
Chemical structures of (**a**) AM, (**b**) PVP, (**c**) HPMC, and (**d**) eudragit.

AM (MW = 410.5 g/mol) was purchased from Chengdu Biopurify Phytochemicals (Shincuan, China), while HPMC, PVP, and eudragit were purchased from Merck (Darmstadt, Germany). The chemical structures of AM, HPMC, PVP, and eudragit are shown in Figure 1. All chemicals were used as received without further purification.

### 2.2. PXRD Measurement

The PXRD patterns were collected using a Kristalloflex Diffractometer D500 (Siemens, Berlin, Germany) with the following conditions: target Cu, filter Ni, voltage 40 kV, current 30 mA, scanning rate 0.75°/min, and scanning angle of 2θ = 10°–40°.

### 2.3. Crystalline Solubility Measurements

The crystalline solubility of AM was determined using 50 mM phosphate buffer at pH 7.4 and 25 °C containing 3% (*v*/*v*) DMSO. Each polymer was dissolved in the phosphate buffer with various concentrations, and excessive crystalline AM powder was added to the solution of the polymer. Subsequently, the solutions were shaken for 48 h and filtered through a 0.45-μm membrane filter, diluted with acetonitrile, and determined using HPLC.

### 2.4. Nucleation Induction Time Measurements

Each polymer was dissolved in 50 mM phosphate buffer at pH 7.4 at a concentration of 500 μg/mL. The AM-supersaturated solutions were prepared by adding a stock solution of AM (1500 μg/mL in DMSO) to the polymer solutions, leading to a final DMSO concentration of 2% (*v*/*v*) at 25 °C. The solutions were stirred at 150 rpm at 25 °C, filtered through a 0.45-μm membrane filter at different time points, diluted with acetonitrile, and determined using HPLC.

### 2.5. HPLC Conditions

HPLC was analyzed using a Dionex-Ultimate 3000 HPLC (Dionex, Sunnyvale, CA, USA) equipped with an Inertsil ODS C18 (6.0 × 150 mm) column at 30 °C. The mobile was composed of acetonitrile and 0.1% formic acid in water at a ratio of 95:5. The samples were analyzed by injecting 10 μL, and the flow rate was set to 1 mL/min detected at 244 nm using a UV detector. Standard solutions of 1, 5, 10, 25, 50, and 100 μg/mL were prepared in the mobile phase. The linear coefficient of determination (R^2^) value of AM standard solution was 0.9996.

### 2.6. FT-IR Spectroscopy

Each sample conducted in Nucleation Induction Time Measurements was evaluated using a Nicolet iS5 FT-IR spectrometer (Thermo Scientific, Waltham, MA, USA) to evaluate the interaction of AM with each polymer in an aqueous solution (on 32 mm). The FT-IR spectra of each sample in an aqueous solution were obtained by subtracting the FT-IR spectra of water as a blank value from that of each sample in the aqueous solution.

### 2.7. NMR Measurements

Each polymer was dissolved in D_2_O, and the AM-supersaturated solutions were prepared by adding a stock solution of AM in DMSO-d6 to the additive solutions at 25 °C. The sample solution was measured after stirring at 150 rpm at 25 °C for one minute using Bruker 500 MHZ NMR spectrometer (Billerica, MA, USA).

### 2.8. In Silico Study

AM’s interactions with water-soluble chitosan were examined using a computer with the following specifications: Intel(R) Celeron(R) N4020 CPU @ 1.10 GHz, installed RAM 8.00 GB, 64-bit operating system, ×64-based processor, Chemdraw, Discovery Studio v. 16.1.0 free trial (Dassault Systemes BIOVIA, San Diego, CA, USA), and Autodock Tools (ADT) 1.5.6 software. AM and polymers were downloaded in two-dimensional (2D) type from Pubchem.ncbi.mlm.gov, or the structures were drawn using Chemdraw 2D, and the energy was reduced using MM2+ on Chemdraw 3D software. The structures were loaded into the Discovery Studio software version 16.1.0 (Dassault Systemes BIOVIA, Waltham, MA, USA) and saved as a PDB file. Subsequently, the interaction was observed using by ligand-ligand interaction method to obtain the hydrogen bonding, binding energy, and distance of each interaction between AM and polymer.

### 2.9. Viscosity Test

Viscosity was measured using an Ametek Brookfield DVE viscometer with a volume sample of ±200 mL. The size of the spindle used is No. 61, with a rotation speed of 100 rpm.

## 3. Results

**Figure 2:**
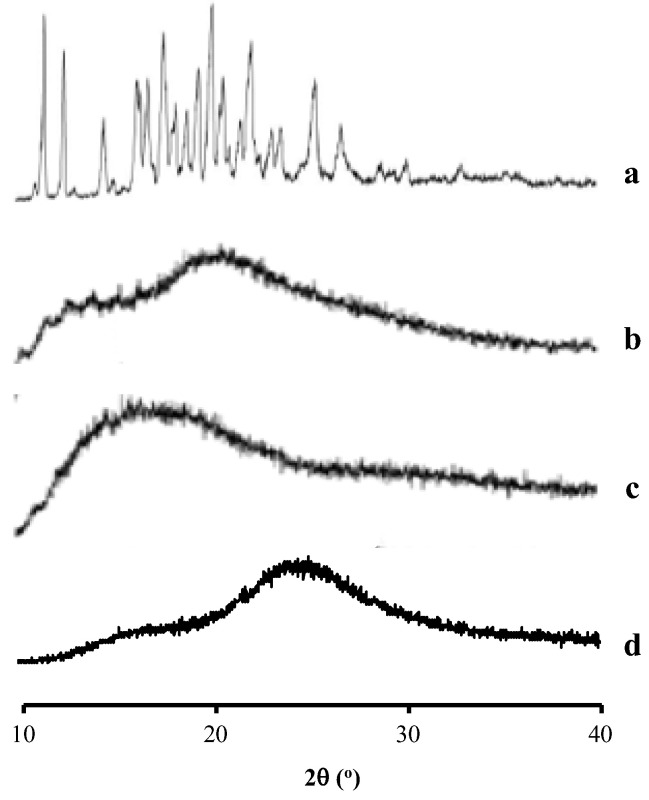
Characteristic diffraction peaks in PXRD of (**a**) AM, (**b**) PVP, (**c**) eudragit, and (**d**) HPMC.

From Figure 2, the AM showed the characteristic diffraction peaks in the PXRD pattern, indicating its crystalline state. Meanwhile, HPMC, PVP, and eudragit showed a halo pattern without any diffraction peaks. This indicated that the polymers used were in an amorphous state.

### 3.1. Crystalline Solubility Measurements

**Figure 3:**
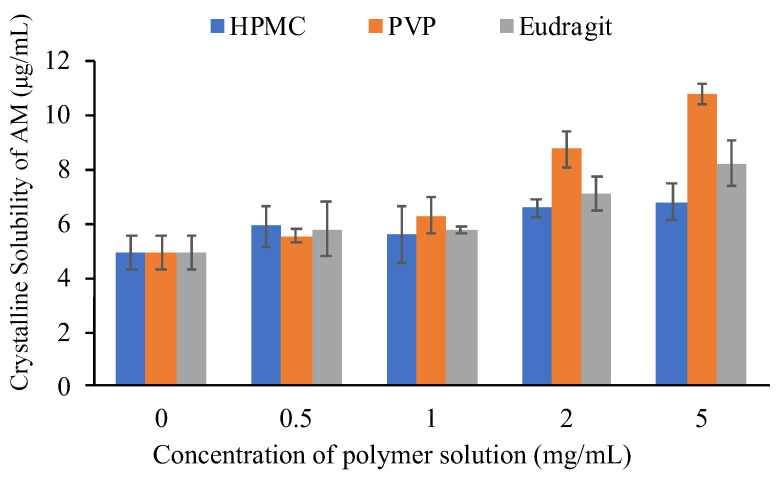
Crystalline solubility of AM at 25 °C in the polymer solutions with various concentrations.

The equilibrium solubilities of AM were determined in the absence and presence of selected polymers with various concentrations to evaluate the effect of each polymer on the crystalline solubility of AM. In this study, the solubility of AM crystal in the presence of selected polymers with various concentration are summarized in Figure 3. The equilibrium solubility values of crystalline AM at 50 mM phosphate buffer at pH 7.4 and 25 °C, where the AM molecule exists in the un-ionized form, was 4.9 μg/mL. This indicated that AM has extremely poor aqueous solubility. An increase in the equilibrium solubility of AM in the presence of an additive was observed, specifically at the polymer concentration of 2 mg/mL. The results suggested that the prolonged nucleation induction time of AM in polymers solutions was caused by the inhibitory effect of polymers on AM crystal nucleation. Furthermore, the micelle formation of polymers contributed to the increase in thermal equilibrium solubility of AM in the polymer solutions. The concentration of AM in the PVP solution was also higher compared to others (eudragit and HPMC). This indicated that the inhibitory effect of PVP on AM crystal nucleation would be stronger than eudragit and HPMC.

**Figure 4:**
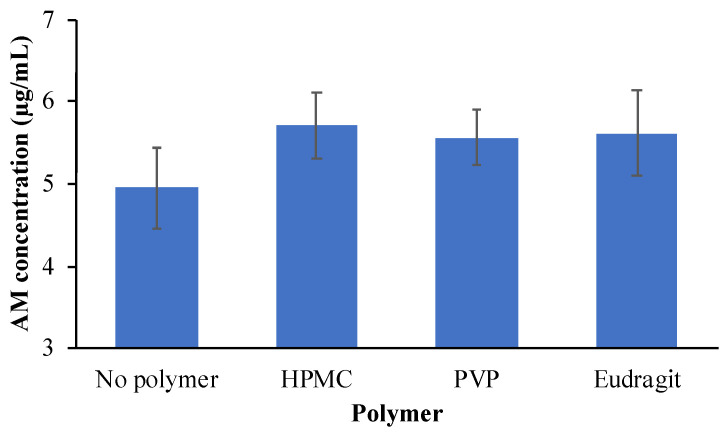
Crystalline Solubility of AM at 25 °C in each polymer solution at the concentration of 500 μg/mL.

A previous report stated that an increase in the equilibrium solubility of a drug by the presence of polymers could decrease the apparent supersaturation level of drugs [10]. In this study, HPMC, PVP, and eudragit had little effect on the thermal equilibrium solubility of AM at a concentration of 500 μg/mL, as shown in Figure 4. At this polymer concentration, the effect of polymers on the equilibrium solubility of the AM can be negligible. Therefore, the polymer concentration of 500 μg/mL was selected for the induction time measurement to prevent the decrease of the apparent supersaturation level from AM.

### 3.2. Nucleation Induction Time Measurements

**Figure 5:**
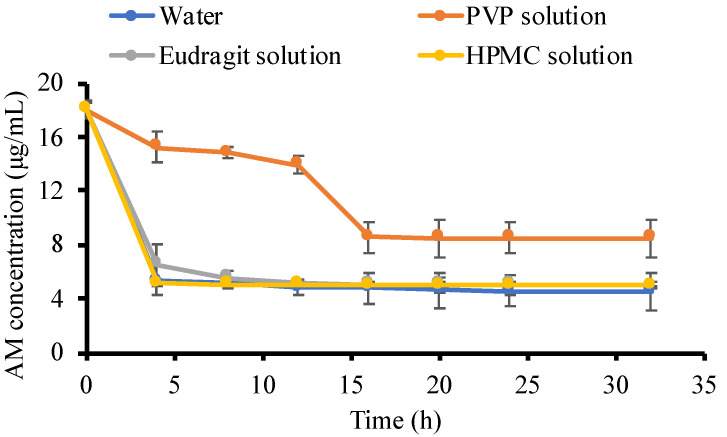
The concentration of AM in the phosphate buffer at pH 7.4 and each polymer solution *n* = 3, mean ± standard deviation).

The crystallization tendency of the drug from the solution can be inferred from their nucleation induction times. The induction time was determined by plotting the concentration of the drug versus time. Figure 5 revealed the concentration of AM in the supersaturated solutions with and without the presence of polymers (500 μg/mL). The initial concentration of the AM was set at 20 μg/mL, which was approximately 4–5 times higher than the crystalline solubility of CLT (4.9 μg/mL). In the phosphate buffer, the AM concentration immediately decreased to AM thermal equilibrium solubility within 4 h. This indicated that the AM quickly formed crystal nuclei in the supersaturated solution, followed by crystal growth. A similar result was also observed in the addition of HPMC and eudragit, where the AM rapidly recrystallized in both solutions. This indicated that the ability of HPMC and eudragit to inhibit AM crystal nucleation was weak. Meanwhile, PVP maintained the initial supersaturation level of AM within 4 h, and the concentration in the PVP solution started to decrease after 12 h, indicating the strong ability to inhibit AM crystal nucleation.

**Figure 6:**
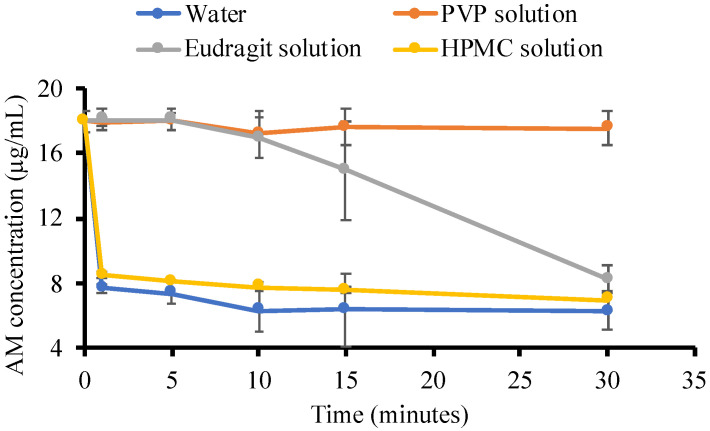
The concentration of AM in the water and each polymer solution for 30 min (*n* = 3, mean ± standard deviation).

The nucleation induction time was evaluated at the beginning of measurement (1, 5, 10, 15, and 30 min), as shown in Figure 6, to confirm the ability of each polymer to inhibit AM crystal nucleation. In the phosphate buffer, the AM concentration rapidly decreased after 1 min, indicating that AM has a high crystallization tendency in the solution. AM also crystallized rapidly in the HPMC solution after 1 min. Furthermore, a high concentration of AM was maintained for 15 min in the eudragit solution but rapidly decreased due to rapid crystallization. This suggested that eudragit can inhibit the AM crystal nucleation, although it has a weak ability. Based on these induction times, it is apparent that the rate of nucleation of AM from PVP solutions was slower compared to HPMC and eudragit due to the strong ability of PVP in the inhibition of AM crystal nucleation.

### 3.3. FT-IR Spectroscopy Analysis

**Figure 7:**
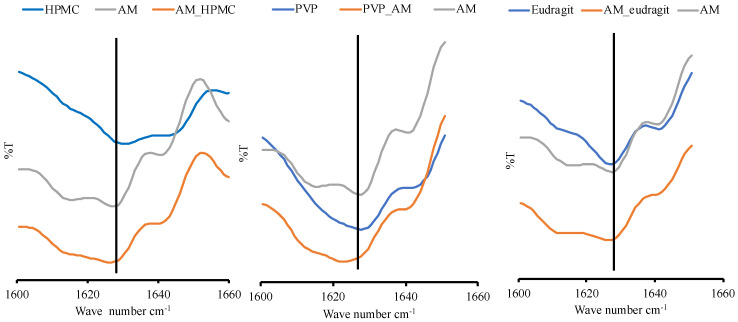
FT−IR spectrum of each sample in the range of 1600–1660 cm^−1^.

Solution FT-IR spectra in water are not easy to obtain as water absorbs strongly throughout most of the IR region. Therefore, the FT-IR spectrum of pure water was subtracted from the FT-IR spectrum of each sample. This was carried out to obtain the FT-IR spectra in an aqueous solution, as shown in Figure 7. The spectra of the carbonyl group of AM and PVP measured in an aqueous solution were observed at around 1627.63 cm^−1^ and 1628.59 cm^−1^, respectively. The carbonyl group spectra of AM in AM-PVP solution were shifted to around 1623.77 cm^−1^. This is due to the possible intermolecular interaction between the carbonyl group of AM as an acceptor of hydrogen with PVP as a donor of hydrogen. However, the carbonyl group spectra of eudragit and AM-eudragit were found at around 1627.63 cm^−1^ and 1626.66 cm^−1^, respectively. This shows the possible weak intermolecular interaction or no interaction between AM and eudragit. The carbonyl group of AM in the HPMC solution was observed at around 1626.66 cm^−1^. These results suggested that the interaction between AM and HPMC was not detected compared to the nucleation time measurement. The slight shift observed in AM-HPMC can be due to the formation of a homogenous mixture between AM and HPMC. Although the shift in spectra in the AM-PVP solution was observed, the spectra were not clear enough because the concentration of all samples was very low. Therefore, further investigation of AM-PVP is still needed to clarify the effect of the interaction of AM-PVP on the supersaturated solution of AM.

### 3.4. NMR Analysis

**Figure 8:**
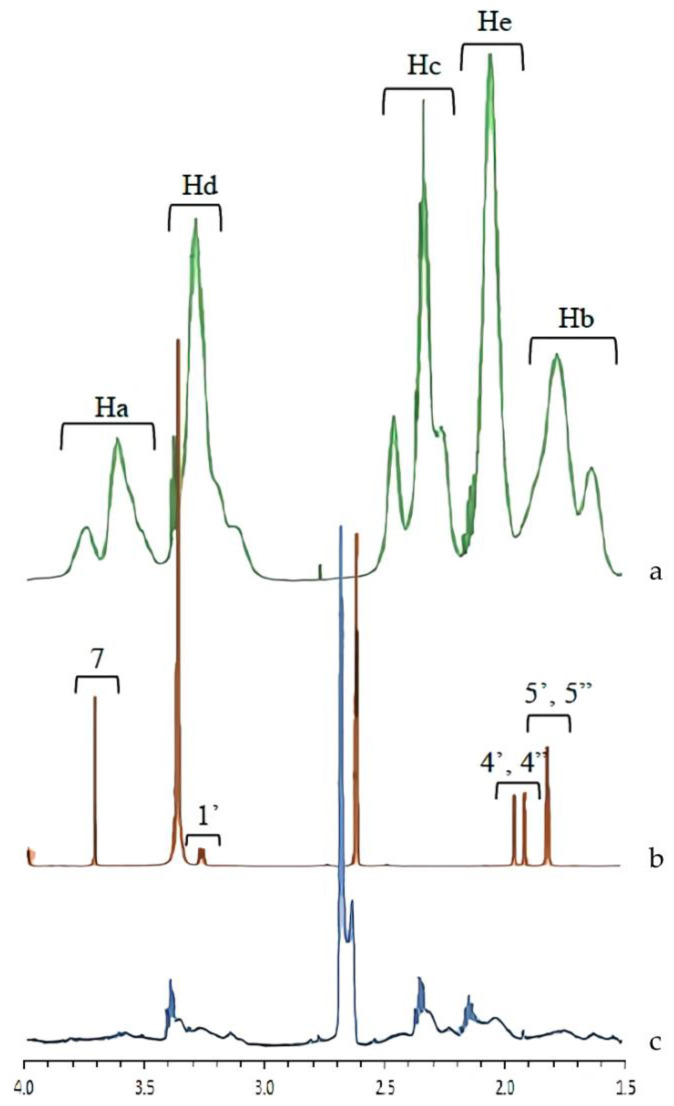
Full ^1^H NMR spectra of (**a**) PVP, (**b**) AM, and (**c**) AM-PVP peak region in the PVP solution.

To confirm the interaction between AM and PVP in the supersaturated solution, ^1^H NMR was also performed. Figure 8 shows the ^1^H NMR spectra of the AM-saturated solutions in the presence of PVP, where the peak assignment spectra were assigned as described in the previous studies [10,22]. The AM peaks were significantly broadened compared with those observed in the buffer. NMR peak width and broadening were attributed to molecular mobility and suppression. Therefore, AM mobility was suppressed in the presence of PVP. The peaks PVP were shifted upfield by the addition of AM, specifically Ha and Hc peaks. The chemical shifts of ^1^H PVP and differences in each sample are summarized in Table 1. These results suggested that the methyl group from PVP formed intermolecular interactions with AM in the supersaturated solution, leading to mobility suppression and the maintenance of AM supersaturation for a long time.

### 3.5. Viscosity Measurement

The viscosity was measured to evaluate the effect of an increase in the viscosity of polymer solution, leading to a decrease in the mobility of the drug in supersaturated solutions. The results of the viscosity measurement of each sample are shown in Table 2.

Although the polymer used in this study slightly increased the viscosity of each solution, the difference in the viscosities between solutions with and without polymer was not significant. Therefore, the difference was negligible, indicating that the effect of the solution viscosity on the crystallization inhibition of AM was minimal. This showed that factors other than viscosity contribute to the mobility suppression of AM in PVP solutions. The crystallization inhibition of AM by the supersaturated solution can also be attributed to the interaction between AM and PVP.

### 3.6. In Silico Study

**Figure 9:**
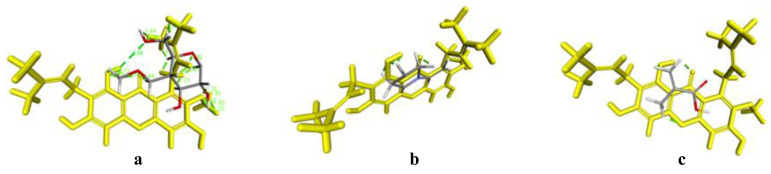
3D Visualization of AM with (**a**) HPMC, (**b**) PVP, and (**c**) eudragit.

**Figure 10:**
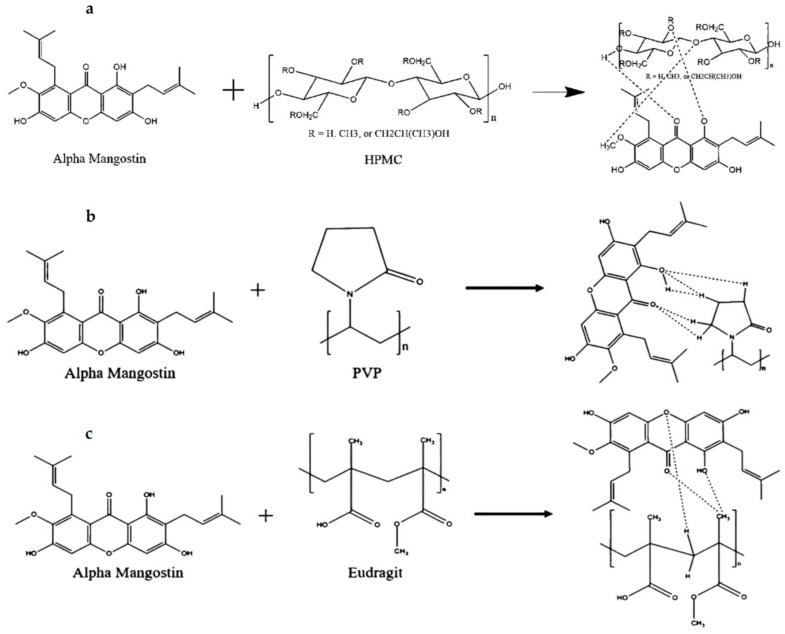
2D Visualization of AM with (**a**) HPMC, (**b**) PVP, and (**c**) eudragit.

The interaction between AM and each polymer was predicted by the ligand-ligand method using Software Discovery Studio Visualizer and Autodock Tools. This was carried out to determine the interaction formation between AM and each polymer by considering the binding energy (Ei) and distance of each contact formed between AM and each polymer. The results of the in silico study are shown in Figure 9 and Figure 10 below.

The results of the visualization screening showed that there is a hydrogen bond formed between AM and the HPMC polymer. The carbonyl and hydrocarbon groups of each ligand become potential proton acceptors and donors. The formation of hydrogen bond interaction can be observed in the carbonyl group of the AM with the hydrocarbon group of HPMC. Based on the interaction distance, it was discovered that the interaction has a distance of 1.75 Å with a bond energy of −4.6 kcal/mol. These data were not in line with the result of induction time measurement, where the AM concentration rapidly decreased in the HPMC solution indicating no interaction between AM and HPMC. Meanwhile, in the mixture of AM and PVP, the interaction of the hydrogen bonds on the carbonyl group of AM with the hydrocarbon group of PVP polymer was observed. Based on the visualization screening, the interaction distance had a value of 0.52 Å and 1.54 Å with a bond energy of −1.5 kcal/mol. Similar to AM with eudragit, the hydrogen bonding interactions were observed in the carbonyl group of AM and the hydrocarbon group of eudragit at a distance of 1.75 Å with a bond energy of −0.9 kcal/mol.

The bond energy value is directly proportional to the activation energy value of the chemical interaction. Therefore, a low bond energy value indicates reduced activation energy. A low distance value will also provide stable and strong bond properties. Based on these results, it can be predicted that a spontaneous reaction and intermolecular interaction between AM and the polymer used in this study will occur due to their energy values and bond distances.

In the case of the PVP and eudragit, the results obtained in the in silico study correspond to that of the FT-IR measurement, where carbonyl group spectra of AM were shifted at around 1350–1750 cm^−1^, indicating their intermolecular interaction. These data supported that the interaction between AM and PVP, as well as eudragit, can maintain the AM in a supersaturated state and inhibit the nucleation with crystal growth of AM, despite their different abilities. This showed that an in silico study could be used as supporting data and initial hypotheses to state the formation of amorphous solid dispersion between AM, PVP, and eudragit. However, the data were not used in the HPMC case, which cannot maintain the initial supersaturation level of AM. A previous study reported that the drug and HPMC easily passed through phase separation in the presence of water [23]. This indicated that absorbed water would accelerate the phase separation of AM and HPMC in the supersaturated solution.

## 4. Discussion

Crystallization inhibition of drugs in a supersaturated solution is an essential component of the drug delivery strategy to enhance mass transport across a biological membrane [8,11]. This showed that polymers need to be selected arbitrarily, and the effectiveness of the polymer used in this study seems to be linked to the properties of the crystallizing solute. The properties and the inhibition ability of the polymer can be key parameters in determining the impact of the polymer on the nucleation rate of drugs. From a theoretical perspective, the ability of the polymer to mix with prenucleation drug aggregates is expected to influence the effectiveness of the polymer in nucleation inhibition. It was assumed that polymers could prevent nucleation by interacting favorably with the solute. A previous study also stated that the strong affinity of the polymer with the solute ensures the interaction between the polymer as well as the solute aggregates and also prevents the reorganization of the solute clusters [24].

**Figure 11:**
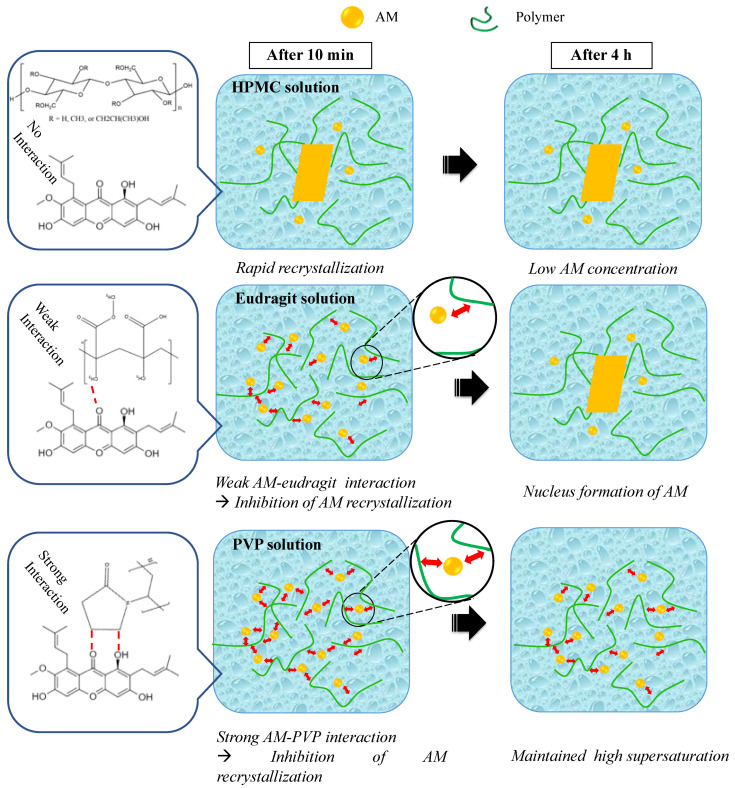
Schematic illustration of crystallization inhibition of AM in each polymer solution.

In this study, the mechanism of each polymer in maintaining the supersaturation levels of AM in a supersaturated solution is discussed, as shown in Figure 11. Based on the induction nucleation time in Figure 6, it was discovered that the concentration of AM rapidly decreased at the beginning of the measurement. This indicated that AM has a high recrystallization tendency in the supersaturated solution. A comparison of the HPMC, PVP, and eudragit systems demonstrated that the AM−polymer interaction and mobility suppression of AM by the polymer became stronger in the order of HPMC, eudragit, and PVP. In the HPMC solution, AM also decreased rapidly until the equilibrium solubility of AM crystal, which produced a similar result. This indicated that the ability of HPMC on nucleation inhibition of AM in the supersaturated solution was limited. A previous report stated that the drug and HPMC could easily pass through phase separation in the presence of water [23]. Therefore, it was assumed that the slight shift observed in AM-HPMC in the FT-IR measurement could be due to the formation of a homogenous mixture between AM and HPMC without any intermolecular interaction. The absorbed water will accelerate the phase separation of AM and HPMC in the supersaturated solution. In the eudragit solution, the high concentration of AM was maintained at the beginning of the induction time measurement. This showed that the AM-eudragit interaction in the supersaturated solution could inhibit the nucleus formation and the subsequent recrystallization of AM. The proton of the methyl group from eudragit also interacted with the carbonyl group of AM in the supersaturated solution, as observed in the FT-IR measurement and the in silico study. However, the high concentration of AM gradually decreased after 15 min in the eudragit solution due to a large amount of water that easily approached the carbonyl group of AM and eudragit. This promotes the phase separation between AM and eudragit, leading to the failure of maintaining the high concentration of AM for a long time. In the case of the PVP solution, AM-PVP showed stronger interactions compared to the other polymers (HPMC and eudragit). The high concentration of AM was maintained for 4 h, although the AM concentration gradually decreased, which can be promoted over time because of the AM crystal growth in the PVP solution. The carbonyl and the methyl groups of AM interacted with the hydrocarbon of PVP in the supersaturated solution, leading to the suppression of the nucleus formation and the subsequent recrystallization of AM. Moreover, the interaction of the methyl group from PVP with the carbonyl group of AM was also observed through NMR measurement.

The results showed that the strong interaction of AM-PVP suppressed the AM to start gathering into clusters induced by water because the part of AM was occupied by hydrogen bond interaction between AM and PVP. Therefore, a large amount of water cannot promote the phase separation between AM and PVP, causing a delay in the phase separation and maintaining the high supersaturation of AM. It was suggested that the difference in the inhibition of AM nucleus formation and the subsequent recrystallization at the beginning of the induction time measurement could be attributed to the variation in each polymer to suppress the clusters of AM to reach a critical size to become stable nuclei due to the difference of strength intermolecular interaction between AM and each polymer. There is also a need to consider the contribution of increased viscosity from each polymer solution to the maintenance of AM supersaturation. A previous investigation reported that the molecular mobility suppression of drugs occurred due to an increase in the viscosity caused by polymer addition in the solution, leading to the maintenance of AM supersaturation. Meanwhile, this study demonstrated that the viscosity of the buffer solution was not significantly increased by the addition of the polymer. The results also showed that the presence of HPMC did not maintain AM supersaturation, although the viscosity of the HPMC solution was higher compared to other polymers. Therefore, the mobility suppression of AM in PVP solutions, the maintenance of AM supersaturation, and the crystallization inhibition from a supersaturated solution can occur due to the interaction between AM and each polymer without any contribution from the viscosity solution, specifically in the case of eudragit and PVP.

## 5. Conclusions

In this study, the impact of different polymers on the supersaturation profiles of AM and the mechanism for maintaining the supersaturation levels of the drug in the biorelevant dissolution tests were elucidated. The induction time measurement revealed that PVP suppressed AM crystal nucleation by forming interactions with AM in supersaturated solutions. Based on the results, PVP is a more effective polymer compared to eudragit and HPMC in the maintenance of AM supersaturation and the crystallization inhibition of AM from a supersaturated solution. This can be attributed to the intermolecular interaction between the proton of a methyl group from PVP with the carbonyl group of AM, leading to the maintenance of AM supersaturation for a long time. This study provided fundamental information into pharmaceutical formulation development, where the selection of polymers for the design of supersaturated formulations is very important to optimize the oral absorption of poorly water-soluble drugs.

## Figures and Tables

**Table 1:** Chemical shifts of proton PVP in the PVP solutions and the difference in the chemical shift compared to that in the buffer.

Sample	Solution	Chemical Shift (ppm)				Different in the Chemical Shift (ppb)			
Peak		Ha	Hb	Hc	He	ΔHa	ΔHb	ΔHc	ΔHe
PVP	PVP	3.610	1.843	2.192	1.979				
AM+PVP	PVP	3.571	1.845	2.106	1.963	39	2	86	16

**Table 2:** Result of viscosity measurement of each sample.

Sample	Viscosity (cps)
HPMC	6.68 ± 0.04
HPMC-AM Buffer	6.84 ± 0.08 3.82 ± 0.08
PVP	4.42 ± 0.04
PVP-AM	4.64 ± 0.06
Eudragit Eudragit-AM	4.88 ± 0.12 4.86 ± 0.12

## Data Availability

Not applicable.
